# TIGIT Expression on Intratumoral Lymphocytes Correlates with Improved Prognosis in Oral Squamous Cell Carcinoma

**DOI:** 10.3390/biomedicines10123236

**Published:** 2022-12-13

**Authors:** Jonas Eichberger, Silvia Spoerl, Gerrit Spanier, Ramona Erber, Juergen Taxis, Johannes Schuderer, Nils Ludwig, Mathias Fiedler, Felix Nieberle, Tobias Ettl, Carol I. Geppert, Torsten E. Reichert, Steffen Spoerl

**Affiliations:** 1 Department of Cranio-Maxillofacial Surgery, University Hospital Regensburg, 93053 Regensburg, Germany; 2 Department of Internal Medicine 5—Hematology/Oncology, Friedrich-Alexander University Erlangen-Nürnberg, 91054 Erlangen, Germany; 3 Institute of Pathology, University Hospital Erlangen, Friedrich-Alexander-Universität Erlangen-Nürnberg (FAU), Comprehensive Cancer Center Erlangen-EMN, 91051 Erlangen, Germany

**Keywords:** T-cell immunoglobulin and ITIM domain (TIGIT), programmed death-ligand 1 (PD-L1), head and neck squamous cell carcinoma (HNSCC), oral squamous cell carcinoma (OSCC), immune checkpoint, immunotherapy

## Abstract

(1) Background: T-cell immunoglobulin and ITIM domain (TIGIT) is a potential immunotherapeutic target in a variety of malignant entities, and antibody-based treatments are currently under investigation in clinical trials. While promising results were observed in patients with lung cancer, the role of TIGIT in oral squamous cell carcinoma (OSCC) as a biomarker as well as a therapeutic target remains elusive. Therefore, we evaluated the role of TIGIT as a prognostic factor in OSCC. (2) Methods: Here, we describe the results of a retrospective tissue microarray (TMA) OSCC cohort. Using immunohistochemistry, TIGIT expression was correlated with overall and recurrence-free survival (OAS and RFS, respectively). Additionally, in silico analysis was performed based on the TCGA Head and Neck Squamous Cell Carcinoma (HNSCC) cohort in order to correlate patients’ survival with TIGIT and CD274 (encoding for PD-L1) gene expression levels. (3) Results: Database analysis revealed a beneficial outcome in OAS for tumor patients with high intraepithelial CD3-TIGIT-expression (*n* = 327). Hereby, OAS was 53.9 months vs. 30.1 months for patients with lower TIGIT gene expression levels (*p* = 0.033). In our retrospective OSCC-TMA cohort, elevated TIGIT levels on CD3+ cells correlated significantly with improved OAS (*p* = 0.025) as well as distant RFS (*p* = 0.026). (4) Conclusions: This study introduces TIGIT as a novel prognostic factor in OSCC, indicating the improved outcome of OSCC patients relative to their increased TIGIT expression. TIGIT might provide therapeutic implications for future immunotherapy in advanced-stage OSCC patients.

## 1. Introduction

Oral squamous cell carcinoma (OSCC) was responsible for nearly 200,000 cancer-related deaths worldwide in 2019 [1]. This life-threatening disease, which originates from the abnormal epithelium of the oral cavity, is strongly associated with preventable risk factors such as tobacco and alcohol abuse [2]. Surgical treatment is considered the main therapeutic approach of OSCC; however, in earlier stages, monotherapy with radiation can be considered, while surgery in combination with adjuvant radio- or chemoradiotherapy is the main treatment in advanced stages of OSCC [3]. Despite recent advances in therapeutic treatment concepts, the 5-year survival rates of OSCC patients remain poor and hardly surpass 60%, especially in advanced carcinomas [4]. The worst affected individuals are those with recurrent disease, whereby the mortality rate increases drastically to 92% [5].

Over the past two decades, investigating anti-cancer immune responses resulted in the establishment of a variety of antitumor therapy strategies targeting immunoregulatory pathways to ultimately strengthen antitumor immunity. It is well known that malignant cells can evade the endogenous immune response by bypassing so-called immune checkpoints, a process that disrupts T-cell responses and promotes tumor progression [6]. Successful clinical trials in head and neck cancer patients led to the approval of anti-PD-1 antibodies nivolumab and pembrolizumab, as well as the anti-PD-L1 antibody durvalumab [7,8,9]. These novel therapeutic targets promise to overcome current limitations of treatment success in recurrent and/or metastatic head and neck squamous cell carcinoma (HNSCC). However, only about a quarter of HNSCC patients were shown to benefit from anti-PD-1/PD-L1 immunotherapy [10]. The high amount of non-responders is under current investigation and various reasons are discussed, including non-immunological functions of PD-L1 or synergistic effects of PD-L1 with other tumor-promoting pathways, which need to be targeted using combination therapies.

Recently, patients diagnosed with recurrent and/or metastatic non-small cell lung cancer (NSCLC) were shown to significantly benefit from combination therapy with atezolizumab, an anti-PD-L1-antibody, and tiragolumab, an antibody blocking TIGIT (T-cell immunoreceptor with immunoglobulin and tyrosine-based inhibitory motif domains) signaling [11]. TIGIT is an immune receptor with an extracellular immunoglobulin domain, a transmembrane sequence, and an internal ITIM motif; it is expressed by various T-lymphocyte subsets as well as natural killer (NK) cells [12]. Its main function is the suppression of T-cell activity by binding to CD155, which subsequently leads to interleukin-10 (IL-10) production by dendritic cells, resulting in a compromised proliferation of CD4+ and CD8+ T cells [13]. This signaling axis is completed by an activating pathway, which involves CD226, since binding of CD226 to CD155 leads to stimulation of T-cell activity [14]. Moreover, TIGIT directly impedes stimulatory pathways up and downstream of the T-cell receptor (TCR), affecting the extent of T-cell responses [15]. A final important mechanism is the activation of FOXP3+ regulatory T cells (Tregs), leading to the suppression of proinflammatory Th1 and Th17 cells [16]. Thus, these findings suggest an immunosuppressive biological role of TIGIT in the tumor microenvironment (TME).

Analogously, recent research indicates that TIGIT plays a tumor-promoting role in several malignant entities. An analysis of multiple preclinical models of pancreatic cancer revealed that TIGIT contributed to immune evasion [17]. In gastric cancer patients, TIGIT exhausted the antitumorigenic CD8+ T-cell response by depriving them of glucose via CD155 binding [18]. There is abundant evidence that, together with PD-L1, TIGIT and the subsequent activation of the TIGIT/CD155 axis are very effective in silencing immune responses to the tumor. In OSCC, little is known about the exact role of TIGIT. So far, there is no direct TIGIT antibody-based treatment being established in clinical OSCC therapy, and this is also the case for the treatment of HNSCC. However, evaluation of TIGIT expression might be useful in the clinical staging of tumors, as demonstrated in a recent study. TIGIT expression on T cells was associated with higher T and N stages in OSCC [19]. Furthermore, when blocking TIGIT and PD-1 simultaneously in an OSCC mouse model, the antitumor activity of NK cells increased dramatically [20]. However, to our knowledge, there are no data demonstrating a prognostic effect of TIGIT in OSCC patients. Therefore, the aim of our study was to link TIGIT expression and prognostic relevance in the clinical context of OSCC. 

## 2. Materials and Methods

### 2.1. In Silico Analysis

CBioPortal (https://www.cBioPortal.org, accessed on 10 October 2022) is an open web resource providing a large database of cancer genomics. We used the Head and Neck Squamous Cell Carcinoma TCGA Firehose Legacy data, which includes 530 tumor samples to analyze the gene expression levels of *TIGIT* and *CD274* (encoding for PD-L1) mRNA in OSCC. Patients with non-available T-stage were excluded. Primary tumor sites included “oral tongue”, “oral cavity”, “floor of mouth”, “buccal mucosa”, “base of tongue”, “alveolar ridge”, “hard palate”, and “lip”. Only primary tumor samples were investigated, reducing the cohort to a final sample number of 334. Next, the online cohort was queried concerning mRNA expression of either *TIGIT* or *CD274*. The mRNA expression levels in tumor samples were normalized relative to the mRNA contents in diploid tissue (RNA seq V2 RSEM). Since not all samples were referenced to diploid tissue, the final number of samples dropped to 327. Lastly, the patient cohort was divided into two groups based on the median expression of *TIGIT* and *CD274*.

The Tumor Immune Single-cell Hub (TISCH) database (http://tisch.comp-genomics.org, accessed on 17 November 2022), an open-access database, was used to further determine TIGIT expression at the single-cell level based on the OSCC_GSE172577 patient cohort which includes 6 patients with OSCC.

Tumor–Immune System Interactions and Drug Bank Database (TISIDB; http://cis.hku.hk/TISIDB, accessed on 17 November 2022) was used to determine the potential association between TIGIT gene expression levels and lymphocyte infiltration abundances and chemokines. Data were analyzed using Spearman correlation analysis.

### 2.2. Patient Cohort

Our in-house cohort included 229 adult Caucasian patients treated for primary OSCC at the Department of Oral and Maxillofacial Surgery, University Hospital Regensburg, between the years 2003 and 2014. All participants underwent surgical resection of the primary lesion to negative margins as well as neck dissection based on the clinical and radiological findings. All patients were staged according to the 7th edition of the UICC (Union internationale contre le cancer) guidelines [21]. The analysis was performed retrospectively, and clinical as well as histopathological data were retrieved from medical records. Adjuvant treatment was based on the recommendation of the multidisciplinary tumor board, and radiotherapy or chemo-radiotherapy was applied accordingly. Patient characteristics are summarized in Table 1.

### 2.3. Immunohistochemical (IHC) Staining

Tissue microarrays (TMAs) were assembled as previously described [22]. Briefly, TMAs contained formalin-fixed, paraffin-embedded human OSCC tissues and corresponding non-neoplastic mucosal tissues of 229 patients of our in-house OSCC cohort. For each patient, three cores (tumor center, the peripheral invasion front, and the adjacent normal tissue) were included to account for the heterogeneity of the tumor and the TME. To evaluate the percentage of CD3+ T cells expressing TIGIT, first, TMAs were cut into 2 µm-thick serial sections and stained as follows: (1) Hematoxylin and eosin (H&E), (2) CD3 IHC, and (3) TIGIT IHC. For TIGIT IHC, the staining protocol was manually performed using a rabbit monoclonal anti-TIGIT antibody (clone BLR047F; dilution 1:200; ab243903, abcam, Cambridge, UK) with an incubation time of 30 min at room temperature (RT), followed by DAB and counterstaining with hematoxylin. For CD3 IHC staining, the protocol was performed on a Ventana Benchmark Ultra automated platform (Ventana Medical Systems, Inc., Oro Valley, AZ, USA) using the SP7 clone (rabbit; dilution 1:150; Zytomed Systems GmbH, Bargteheide, Germany) with an incubation time of 32 min at 37 °C.

### 2.4. Image Analysis

The stained TMA slides were scanned using the Pannoramic 1000 scanner (3DHistech, Budapest, Hungary) and visualized using the CaseViewer software (Version 2.4; 3DHistech, Budapest, Hungary). The evaluation was performed by a board-certified pathologist (R.E.) blinded to clinicopathological and outcome data visualizing H&E, CD3, and TIGIT scans of each TMA core next to each other. First, H&E slides, stained according to the standard in-house protocol, were reviewed regarding the morphology and growth pattern of OSCC. For each case, the semiquantitative percentage of TIGIT expression within CD3+ T cells was assessed (ranging from 0–100%). Moreover, TIGIT assessment was separately performed for the stromal and intraepithelial tumor areas, respectively. 

### 2.5. Statistical Analysis

Statistical analysis was performed using SPSS26 software (IBM Germany GmbH, Ehningen, Germany). Correlations between clinical data and biomarker expression were calculated using Pearson’s Chi-square test. Univariate survival analysis for overall survival (OAS), disease-free survival (DFS), and disease-specific survival (DSS) was calculated using the Kaplan–Meier method. OAS was defined as the time from diagnosis to death by any cause. DFS was determined as the time from therapy to tumor recurrence or death, whichever occurred first. DSS was considered the time from diagnosis to tumor-related death. Median follow-up was calculated using the reverse Kaplan–Meier method. The survival distributions were compared using the log-rank test. For risk adjustment, multivariate Cox regression was applied. The results were reported with hazard ratios (HRs) and 95% confidence intervals (CIs). The proportional hazard assumptions were evaluated using the method of Grambsch and Therneau [23]. All reported *p*-values are two-sided and only determined as statistically significant if *p* < 0.05.

## 3. Results

### 3.1. Characterization of TIGIT Expression in OSCC

**Figure 1:**
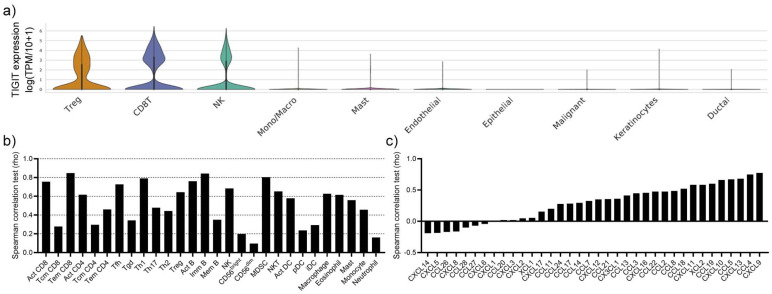
(**a**) Cellular TIGIT gene expression levels in single-cell sequencing data of six patients with OSCC. (**b**) Correlation of TIGIT gene expression levels with immune cell infiltration. (**c**) Correlation between chemokines and the TIGIT gene expression levels.

To evaluate which cells express TIGIT in the TME of OSCC patients, we analyzed a published single-cell sequencing dataset. TIGIT expression was limited to Tregs, CD8+ T cells as well as NK cells, while other immune cell subsets and malignant/non-malignant epithelial cells did not express TIGIT (Figure 1a). Although the expression of TIGIT was limited to these three cell types according to the single-cell sequencing data, the gene expression levels of TIGIT in the bulk sequencing cohort significantly correlated with the infiltration abundances of all tested immune cell subsets, indicating that TIGIT gene expression levels are associated with a greater presence of immune cells in the TME (Figure 1b). Besides the expected significant correlation of TIGIT gene expression levels with CD8+ T cells, Tregs, and NK cells, the infiltration abundances of the immune cell subsets T follicular helper cells (Tfh), type 1 T helper cells (Th1), activated B cells (Act B), immature B cells (Imm B), and myeloid-derived suppressor cells (MDSCs); therefore, cells with immunostimulatory and immunosuppressive functions were most significantly correlated with TIGIT gene expression levels (Figure 1b). To have a possible explanation for the accelerated immune cell infiltration, we correlated TIGIT gene expression levels with chemokine genes. Most chemokines were positively linked to elevated TIGIT gene expression levels, and among these, CXCL9, CCL4, CXCL13, CCL5, and CXCL10 most significantly correlated with TIGIT (Figure 1c). Our data indicate that TIGIT expression is limited to a few cell types in the TME; however, its expression is strongly associated with an upregulation of chemokine expression and an overall increased immune cell infiltration into the tumor tissue.

### 3.2. TIGIT Gene Expression Levels Are Associated with Improved Overall Survival in OSCC

**Figure 2:**
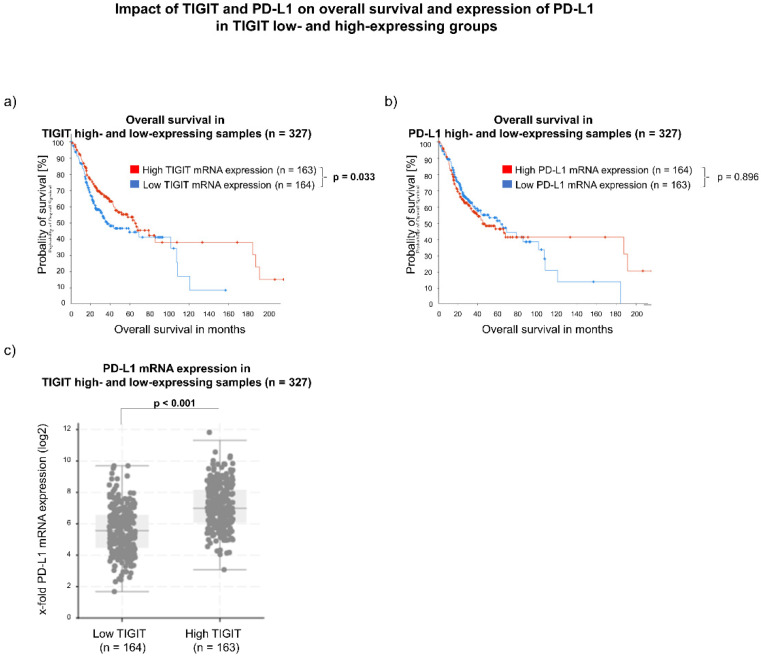
(**a**,**b**) Impact of *TIGIT* and *CD274* mRNA expression levels on overall survival of OSCC patients, generated from the cBioPortal TCGA Firehose Legacy dataset. Groups were divided based on the median. (**c**) An additional analysis of *TIGIT*/*CD274* co-expression demonstrated higher *CD274* levels in patients with high *TIGIT* mRNA expression levels.

The cBioPortal database was used to systematically analyze TIGIT gene expression levels in the tumor tissue of OSCC patients included in the TCGA HNSCC cohort. The in silico cohort characteristics are summarized in Table 1. First, the 327 OSCC samples were divided based on the median of TIGIT mRNA expression. It was defined as low when a z-score ≤ −0.47 was applicable and high if the z-score was >−0.47 (Figure 2). Kaplan–Meier analysis of overall survival in the cohort revealed a significant beneficial impact for patients with high TIGIT gene expression levels. The median OAS for this group was 53.9 months (95% CI: 37.3–159.5 months; *n* = 163), whereas samples with low gene expression levels of TIGIT showed a median OAS of 30.1 months (95% CI: 24.3–100.5 months; *n* = 164; *p* = 0.033). Since anti-TIGIT therapy is clinically combined with anti-PD-L1 therapy, we also evaluated the impact of CD274 gene expression levels on prognosis in the OSCC cohort. Again, groups were selected based on the median, and the outcome was then analyzed using the Kaplan–Meier method. Patients with low CD274 mRNA expression, defined as z-score ≤ −0.45, had a median OAS of 52.3 months (95% CI: 32.8–89.3 months; *n* = 163). Samples abundant of CD274 mRNA, meaning a z-score > −0.45, showed an OAS of 37.3 months (95% CI: 28.0–n/a; *n* = 164; *p* = 0.896). Therefore, CD274 gene expression levels had no significant effect on prognosis in the TCGA OSCC cohort. When analyzing the co-expression of TIGIT and CD274, patients with high TIGIT gene expression levels also displayed a high gene expression level of CD274 (*p* < 0.001) compared to samples with low expression levels of TIGIT.

### 3.3. Expression of TIGIT on CD3+ Cells Correlates with Improved Survival of OSCC Patients

**Figure 3:**
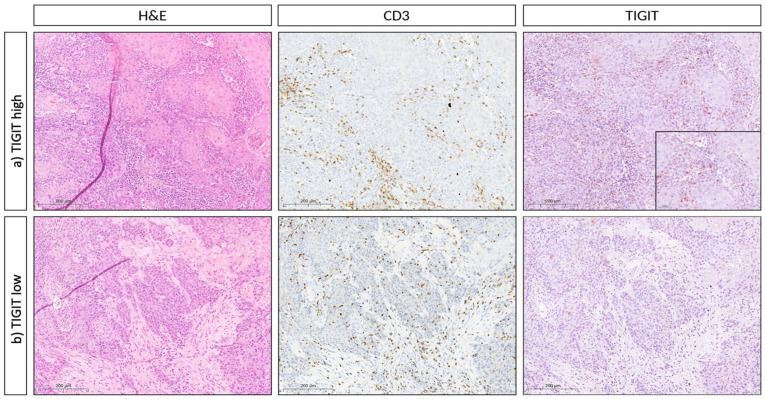
Immunohistochemical TIGIT expression in OSCC TMA cohort. Representative histopathology (hematoxylin & eosin, H&E) and CD3/TIGIT immunohistochemistry (IHC), respectively, of two cases of oral squamous cell carcinoma (OSCC). (**a**) (First row) represents an OSCC with stromal and intraepithelial CD3 and TIGIT double-positive T cells (magnification 400X each, 1000X for the inset). (**b**) (Second row) represents an OSCC with stromal and intraepithelial CD3+ T cells that do not express TIGIT (400X).

To evaluate the TIGIT expression on the protein level, we examined an OSCC TMA and correlated available clinicopathological parameters with TIGIT expression in CD3+ cells. Figure 3 shows hematoxylin & eosin as well as IHC staining (CD3 and TIGIT) in two representative cases. For our analysis, we focused on the tumor specimen with special regard to intraepithelial CD3+ cells. Hereby, higher T-stages were significantly linked to a lower TIGIT expression in these cells, whereas the most frequent T-stage in patients with a lower expression of TIGIT by intraepithelial tumoral CD3+ cells was T1 (Table 2, 37.9%, *p* = 0.047). Additionally, poorly differentiated tumors correlated with lower TIGIT expression levels, whereas in contrast, only one patient with a G3 OSCC was diagnosed with high tumoral CD3+ TIGIT expression (Table 2, *p* = 0.047).

**Figure 4:**
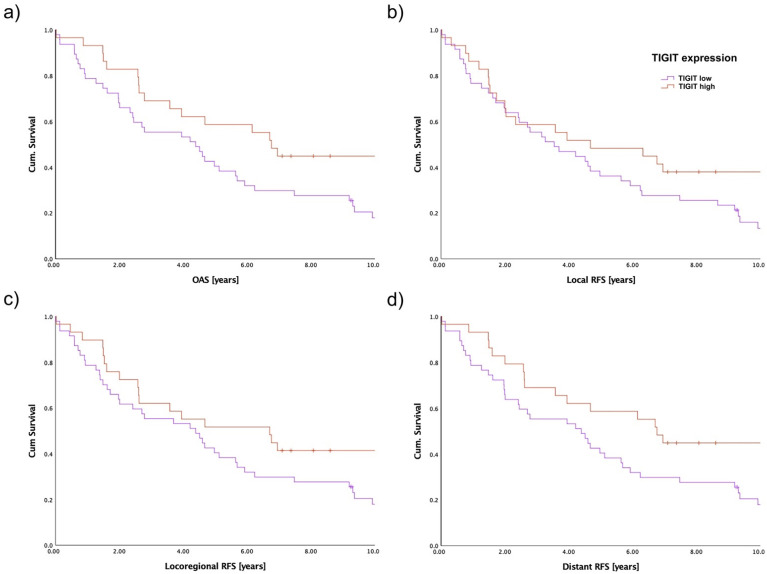
Survival of OSCC patients based on TIGIT expression on CD3+ intratumoral cells assessed by immunostaining of TMA slides. The patient cohort was separated by a median cutoff. (**a**) OAS in the complete cohort (*n* = 76, *p* = 0.025); (**b**) local RFS (*n* = 76, *p* = 0.129); (**c**) locoregional RFS (*n* = 76, *p* = 0.080); (**d**) distant RFS (*n* = 76, *p* = 0.026).

Survival analysis of the present TMA cohort was performed by analyzing OAS as well as RFS using the Kaplan–Meier method. Hereby, local, locoregional, as well as distant RFS of 76 patients were evaluated with regards to TIGIT expression in the population of CD3+ cells (Figure 4). For OAS, patients with a lower intratumoral TIGIT expression in CD3+ cells showed a 5-year survival of 38.3%, whereas tumor patients with overexpression of TIGIT in CD3+ cells displayed a 5-year OAS of 55.2% (*p* = 0.025, Figure 4a). Regarding the evaluation of RFS in detail, no significant differences in the oncologic outcome of both groups in local RFS were observed (*p* = 0.129, Figure 4b). In contrast to local RFS, a trend towards the ameliorated outcome of TIGIT overexpressing patients was evident for locoregional RFS (*p* = 0.080, Figure 4c). When evaluating the survival of OSCC patients with regards to the presence of systemic metastasis, a clearly impaired outcome was observed for patients with lower tumoral TIGIT expression in CD3+ cells, similar to the results of OAS (*p* = 0.026, Figure 4d).

## 4. Discussion

The aim of this study was to characterize the expression levels of TIGIT in OSCC on gene and protein levels and evaluate its prognostic role. Therefore, we systematically searched online databases containing data from OSCC patients. In silico data revealed a significant association of high *TIGIT* mRNA expression levels with high *CD274* mRNA expression levels and favorable OAS. At the protein level, the effect of TIGIT on OAS corroborated with online data. Patients with higher levels of TIGIT on CD3+ cells had a significantly higher 5-year survival rate. Additionally, high TIGIT expression on CD3+ cells was associated with longer RFS concerning distant metastasis.

In terms of the underlying mechanism of TIGIT antibodies, two main antitumorigenic effects are taken into consideration when pharmacologically blocking the TIGIT signaling axis. The main driver of the reinstatement of antitumor activity seems to be the restoration of CD8+ T cells. Despite ambiguous data in the literature, it is generally accepted that a tumor-associated T-cell pool consisting of mainly CD8+ cells is responsible for combating cancer [24]. Results in pre-clinical cancer models show increased activity of CD8+ effector T cells when interfering with TIGIT and PD-1 or PD-L1 simultaneously [18,25]. The second mechanism that was observed was the decrease in regulatory CD4+ T cells or Tregs, respectively. The physiological role of Tregs is the suppression of effector T cell activity, mainly to support autoimmune tolerance and prevent an excessive immune response [26]. In cancer, however, a downregulation of effector T cells is exploited by the tumor to suppress immune activity. It is reported that almost a quarter of tumor-infiltrating lymphocytes (TILs) in the TME are in fact Tregs, while making up only about 5–10% of CD4+ T cells in the bloodstream [27]. In various malignant entities, those Tregs present in the immune microenvironment of the tumor regularly express TIGIT on their cell surface [28,29,30], and it was shown that blockade of TIGIT leads to a significant decrease in Tregs [31,32].

The relevance of TIGIT in HNSCC, the superordinate group of OSCC, is discussed actively in the current literature. Previous studies demonstrated a high expression of TIGIT, PD-L1 and other immune checkpoint molecules in HNSCC [33,34]. Wu et al. also discovered the high expression of TIGIT and its binding partner CD155; the expression on Tregs particularly increased when blocking the PD-1/PD-L1 axis. In a second phase, they used an antibody directed against TIGIT in mice with HNSCC and observed a deceleration of tumor progress, which seemed to be mostly dependent on CD8+ T cell regeneration [35]. Conversely, upon inhibition of TIGIT, PD-L1 was upregulated in the tumor immune microenvironment. A combination of anti-TIGIT and anti-PD-L1 therapy significantly decreased tumor growth and alleviated weight loss in HNSCC-bearing mice [36]. 

When looking at survival data in patients with breast or colorectal cancer, TIGIT expression in the tumors was linked to improved OAS [37]. Extensive analysis of gene databases also revealed a beneficial role of TIGIT in head and neck or breast cancer, while in renal clear cell carcinoma, tumors expressing TIGIT demonstrated poorer patient outcomes [38]. On the other hand, a meta-analysis from 2021 found TIGIT expression in different solid tumors to be negatively associated with survival [39]. These findings demonstrate the controversial role of TIGIT in the clinical setting, which makes it difficult to define TIGIT as a predictive biomarker in clinical practice. Our results confirm the observation of TIGIT to be positively associated with patient survival, which is completely novel for the tumor entity OSCC. While this finding appears to be contradictory at first, and also regarding the ongoing clinical trials using TIGIT as a therapeutic target, we were able to show that TIGIT expression correlates with the increased infiltration abundances of a variety of immune cell subsets. Thus, the favorable prognosis of TIGIT high-expressing tumors might be explained by the overall immune landscape of these tumors, which could potentially be defined as immune “hot tumors”. Hereby, we were able to show that chemokines such as CXCL9, as well as other ligands of CXCR3 (e.g., CXCL10, CXCL11), were highly positively correlated with TIGIT gene expression in OSCC. These CXCR3 ligands could ultimately result in increased tumor immune infiltration. In this regard, the effect of blocking TIGIT on elevated tumor-infiltrating CD3+ cells should be scrutinized in detail: Thus, it could be reasoned that blocking TIGIT affects primarily immune suppressive T cells such as Tregs, more than affecting the functionality of CD8+ cytotoxic T cells. Hereby, the results of Preillon et al. provide an interesting insight into a tumor-specific T-cell function within TIGIT-antibody treatment: TIGIT-expressing TILS were shown to have impaired antitumor activity; however, TIGIT antibody treatment was able to potently reverse these effects in vitro [40]. Beyond this, TIGIT-antibody treatment resulted directly in the depletion of immunosuppressive TIGIT+ Tregs [40], which further supports the antitumor activity of a TIGIT-directed antibody [41,42]. As TIGIT expression is a sign of tumor antigen-specific cytotoxic T cells and TILs, we expect that pharmacological blockade of TIGIT results in an enhanced cytotoxic T cell-mediated immune response. However, this is achieved not only by modulating dysfunctional CD8+ T cells and restoring their anti-tumoral cytotoxic properties but even more by depleting highly suppressive TIGIT+ intratumoral Tregs and enabling further anti-tumoral responses.

Considering these data, TIGIT remains to be extensively studied as a predictive marker and therapeutic agent in the clinic, especially when it comes to differentiating its role in various tumor entities such as OSCC. 

As previously discussed, TIGIT is not only a biomarker but also already targeted by therapeutic antibodies in clinical practice. In early clinical trials, the safety and antitumor activity of TIGIT-antibodies has been affirmed. However, large clinical trials combining different tumor entities are still in the process, and data are not definitive yet. The CITYSCAPE trial delivers compelling arguments for a simultaneous blockade of the PD-1/PD-L1 axis and TIGIT in patients with NSCLC. Patients receiving a TIGIT antibody (tiragolumab) in addition to PD-L1 blockade had an improved median survival time of about 2 months compared to the group solely receiving a PD-L1 antibody (atezolizumab) [11]. A second multicentric study, using a different TIGIT antibody, vibostolimab, included patients being diagnosed with various advanced solid tumors, including head and neck malignancies. Vibostolimab, together with Pembrolizumab, also revealed a beneficial effect, especially for patients with NSCLC. In this context, side effects such as pyrexia were reported to be acceptable [43]. Another fully humanized TIGIT antibody is the EOS-448, currently being investigated in a first-in-human study [44]. EOS-448 activates TIGIT-low T cells and depletes Tregs as well as „exhausted” TIGIT-high T cells. In this first set of heavily pretreated patients, only one had a partial response, whereas half of the remaining patients demonstrated stable disease, while the other one had progressing cancer. 

More TIGIT antibodies, such as Domvanalimab (AB-154, trial no. NCT03628677), Ociperlimab, Etigilimab, SEA-TGT (trial no. NCT04254107), and ASP8374, are being investigated in early clinical trials including patients with advanced/metastasized solid tumors, relapsing after different prior therapies [36,45,46]. Most of them are used in combination with checkpoint inhibitors. However, also treatment arms with monotherapy are included. Most studies are mainly „basket trials”, combining different tumor entities, also including HNSCCs. A strong focus on TIGIT antibodies targets patients with lung cancer. To date, there are no published studies containing only HNSCCs, so there is no evident data on the efficacy of TIGIT antibodies in head and neck cancer and, furthermore, in OSCC. Nevertheless, as multiple clinical trials investigating different TIGIT antibodies are ongoing, we will obtain comprehensive data in the future in order to determine which TIGIT antibody acts most efficiently in head and neck cancer patients and even more in the subgroup of OSCC. 

## 5. Conclusions

Taken together, our data indicate a relevant prognostic role for TIGIT as a biomarker in OSCC patients. Based on our findings, clinical treatment with TIGIT antibodies is quite promising in OSCC patients, a group of patients with limited therapeutic opportunities and a strong need for novel advances in antitumor therapy.

## Figures and Tables

**Table 1:** Clinicopathological characteristics of the in silico cohort (*n* = 327).

Characteristic		Count	%
Age at diagnosis		19–90 years; median 61 years	−
Sex	Female	101	32.0%
	Male	225	68.0%
Positive history of nicotine abuse	No	78	23.9%
	Yes	247	74.9%
	N/A	2	1.2%
Positive history of alcohol abuse	No	107	32.7%
	Yes	219	67.0%
	N/A	1	0.3%
Tumor site	Tongue	129	39.4%
	Oral cavity	67	20.5%
	Floor of mouth	59	18.1%
	Base of tongue	23	7.0%
	Buccal mucosa	22	6.7%
	Alveolar ridge	17	5.2%
	Hard palate	7	2.1%
	Lip	3	1.0%
Tumor size	T1	33	10.1%
	T2	101	30.9%
	T3	68	20.8%
	T4	11	3.4%
	T4a	95	29.0%
	T4b	4	1.2%
	Tx	15	4.6%
Cervical node stage	N0	168	51.5%
	N1	60	18.4%
	N2a	45	13.9%
	N2b	25	7.7%
	N2c	11	3.5%
	N3	7	2.1%
	Nx	4	1.2%
	N/A	7	2.1%
Tumor grade	G1	48	14.5%
	G2	201	61.1%
	G3	65	19.6%
	G4	4	2.1%
	Gx	6	1.7%
	N/A	3	1.0%

**Table 2:** Patients’ clinicopathological characteristics according to TIGIT expression of CD3+ cells (median cutoff; *n* = 76).

		χ2 TIGIT Median Cut-Off				
		≤Median		>Median		*p*
		Count	%	Count	%	
Age at diagnosis	<70.0	31	66.0%	24	82.8%	0.112
	≥70.0	16	34.0%	5	17.2%	
Sex	Female	15	31.9%	12	41.4%	0.402
	Male	32	68.1%	17	58.6%	
Positive history of nicotine abuse	No	12	25.5%	5	17.2%	0.399
	Yes	35	74.5%	24	82.8%	
Positive history of alcohol abuse	No	10	21.3%	11	37.9%	0.115
	Yes	37	78.7%	18	62.1%	
Tumor size	T1	6	12.8%	11	37.9%	**0.047**
	T2	22	46.8%	8	27.6%	
	T3	5	10.6%	1	3.4%	
	T4a	14	29.8%	9	31.0%	
	T4b	0	0.0%	0	0.0%	
Cervical node status	N0	23	48.9%	16	55.2%	0.795
	N1	8	17.0%	4	14.0%	
	N2a	2	4.3%	0	0.0%	
	N2b	9	19.1%	5	17.2%	
	N2c	5	10.6%	4	14.0%	
	N3a	0	0.0%	0	0.0%	
	N3b	0	0.0%	0	0.0%	
Tumor grade	G1	0	0.0%	2	6.9%	**0.047**
	G2	39	83.0%	26	89.7%	
	G3	8	17.0%	1	3.4%	
	G4	0	0.0%	0	0.0%	
Lymph vessel invasion	L0	35	74.5%	24	82.8%	0.399
	L1	12	25.5%	5	17.2%	
Vessel invasion	V0	44	93.6%	27	93.1%	0.930
	V1	3	6.4%	2	6.9%	
Perineural invasion	Pn0	46	97.9%	27	93.1%	0.300
	Pn1	1	2.1%	2	6.9%	
Adjuvant therapy	No	21	44.7%	12	41.4%	0.931
	Radiotherapy	19	40.4%	13	44.8%	
	Radiochemotherapy	7	14.9%	4	13.8%	
	total	47	100.0%	29	100.0%	

## Data Availability

Data can be obtained by scientists that work independently from the industry on request. Data are not stored on publicly available servers.

## References

[1] Kocarnik J.M., Compton K., Dean F.E., Fu W., Gaw B.L., Harvey J.D., Henrikson H.J., Lu D., Pennini A., Global Burden of Disease 2019 Cancer Collaboration (2022). Cancer Incidence, Mortality, Years of Life Lost, Years Lived with Disability, and Disability-Adjusted Life Years for 29 Cancer Groups from 2010 to 2019: A Systematic Analysis for the Global Burden of Disease Study 2019. JAMA Oncol..

[2] Kumar M., Nanavati R., Modi T.G., Dobariya C. (2016). Oral cancer: Etiology and risk factors: A review. J. Cancer Res. Ther..

[3] Chow L.Q.M. (2020). Head and Neck Cancer. N. Engl. J. Med..

[4] Güneri P., Epstein J.B. (2014). Late stage diagnosis of oral cancer: Components and possible solutions. Oral Oncol..

[5] Weckx A., Riekert M., Grandoch A., Schick V., Zöller J.E., Kreppel M. (2019). Time to recurrence and patient survival in recurrent oral squamous cell carcinoma. Oral Oncol..

[6] Ferris R.L. (2015). Immunology and Immunotherapy of Head and Neck Cancer. J. Clin. Oncol..

[7] Ferris R.L., Blumenschein G., Fayette J., Guigay J., Colevas A.D., Licitra L., Harrington K., Kasper S., Vokes E.E., Even C. (2016). Nivolumab for Recurrent Squamous-Cell Carcinoma of the Head and Neck. N. Engl. J. Med..

[8] Burtness B., Harrington K.J., Greil R., Soulières D., Tahara M., de Castro G., Psyrri A., Basté N., Neupane P., Bratland Å. (2019). Pembrolizumab alone or with chemotherapy versus cetuximab with chemotherapy for recurrent or meta-static squamous cell carcinoma of the head and neck (KEYNOTE-048): A randomised, open-label, phase 3 study. Lancet.

[9] Ferris R., Haddad R., Even C., Tahara M., Dvorkin M., Ciuleanu T., Clement P., Mesia R., Kutukova S., Zholudeva L. (2020). Durvalumab with or without tremelimumab in patients with recurrent or metastatic head and neck squamous cell carcinoma: EAGLE, a randomized, open-label phase III study. Ann. Oncol..

[10] Botticelli A., Cirillo A., Strigari L., Valentini F., Cerbelli B., Scagnoli S., Cerbelli E., Zizzari I.G., Rocca C.D., D’Amati G. (2021). Anti-PD-1 and Anti-PD-L1 in Head and Neck Cancer: A Network Meta-Analysis. Front. Immunol..

[11] Cho B.C., Abreu D.R., Hussein M., Cobo M., Patel A.J., Secen N., Lee K.H., Massuti B., Hiret S., Yang J.C.H. (2022). Tiragolumab plus atezolizumab versus placebo plus atezolizumab as a first-line treatment for PD-L1-selected non-small-cell lung cancer (CITYSCAPE): Primary and follow-up analyses of a randomised, double-blind, phase 2 study. Lancet Oncol..

[12] Yu X., Harden K., Gonzalez L.C., Francesco M., Chiang E., Irving B., Tom I., Ivelja S., Refino C.J., Clark H. (2008). The surface protein TIGIT suppresses T cell activation by promoting the generation of mature immunoregulatory dendritic cells. Nat. Immunol..

[13] Chauvin J.-M., Pagliano O., Fourcade J., Sun Z., Wang H., Sander C., Kirkwood J.M., Chen T.-H.T., Maurer M., Korman A.J. (2015). TIGIT and PD-1 impair tumor antigen–specific CD8+ T cells in melanoma patients. J. Clin. Investig..

[14] Bottino C., Castriconi R., Pende D., Rivera P., Nanni M., Carnemolla B., Cantoni C., Grassi J., Marcenaro S., Reymond N. (2003). Identification of PVR (CD155) and Nectin-2 (CD112) as Cell Surface Ligands for the Human DNAM-1 (CD226) Activating Molecule. J. Exp. Med..

[15] Joller N., Hafler J.P., Brynedal B., Kassam N., Spoerl S., Levin S.D., Sharpe A.H., Kuchroo V.K. (2011). Cutting Edge: TIGIT Has T Cell-Intrinsic Inhibitory Functions. J. Immunol..

[16] Joller N., Lozano E., Burkett P.R., Patel B., Xiao S., Zhu C., Xia J., Tan T.G., Sefik E., Yajnik V. (2014). Treg Cells Expressing the Coinhibitory Molecule TIGIT Selectively Inhibit Proinflammatory Th1 and Th17 Cell Responses. Immunity.

[17] Freed-Pastor W.A., Lambert L.J., Ely Z.A., Pattada N.B., Bhutkar A., Eng G., Mercer K.L., Garcia A.P., Lin L., Rideout W.M. (2021). The CD155/TIGIT axis promotes and maintains immune evasion in neoantigen-expressing pancreatic cancer. Cancer Cell.

[18] He W., Zhang H., Han F., Chen X., Lin R., Wang W., Qiu H., Zhuang Z., Liao Q., Zhang W. (2017). CD155T/TIGIT Signaling Regulates CD8+ T-cell Metabolism and Promotes Tumor Progression in Human Gastric Cancer. Cancer Res..

[19] Liu X., Li Q., Zhou Y., He X., Fang J., Lu H., Wang X., Wang D., Ma D., Cheng B. (2020). Dysfunctional role of elevated TIGIT expression on T cells in oral squamous cell carcinoma patients. Oral Dis..

[20] Patin E.C., Dillon M.T., Nenclares P., Grove L., Soliman H., Leslie I., Northcote D., Bozhanova G., Crespo-Rodriguez E., Baldock H. (2022). Harnessing radiotherapy-induced NK-cell activity by combining DNA damage–response inhibition and immune checkpoint blockade. J. Immunother. Cancer.

[21] Soben L.H., Gospodarowicz M.K., Wittekind C. (2009). TNM Classification of Malignant Tumors.

[22] Kononen J., Bubendorf L., Kallioniemi O., Bärlund M., Schraml P., Leighton S., Torhorst J., Mihatsch M.J., Sauter G., Kallionimeni O.-P. (1998). Tissue microarrays for high-throughput molecular profiling of tumor specimens. Nat. Med..

[23] Grambsch P.M., Therneau T.M. (1994). Proportional hazards tests and diagnostics based on weighted residuals. Biometrika.

[24] Balermpas P., Rödel F., Rödel C., Krause M., Linge A., Lohaus F., Baumann M., Tinhofer I., Budach V., Gkika E. (2015). CD8+ tumour-infiltrating lymphocytes in relation to HPV status and clinical outcome in patients with head and neck cancer after postoperative chemoradiotherapy: A multicentre study of the German cancer consortium radiation oncology group (DKTK-ROG). Int. J. Cancer.

[25] Hung A.L., Maxwell R., Theodros D., Belcaid Z., Mathios D., Luksik A.S., Kim E., Wu A., Xia Y., Garzon-Muvdi T. (2018). TIGIT and PD-1 dual checkpoint blockade enhances antitumor immunity and survival in GBM. Oncoimmunology.

[26] Bettelli E., Carrier Y., Gao W., Korn T., Strom T.B., Oukka M., Weiner H.L., Kuchroo V.K. (2006). Reciprocal developmental pathways for the generation of pathogenic effector TH17 and regulatory T cells. Nature.

[27] Oleinika K., Nibbs R.J., Graham G.J., Fraser A.R. (2013). Suppression, subversion and escape: The role of regulatory T cells in cancer progression. Clin. Exp. Immunol..

[28] Wu K., Zeng J., Shi X., Xie J., Li Y., Zheng H., Peng G., Zhu G., Tang D., Wu S. (2022). Targeting TIGIT Inhibits Bladder Cancer Metastasis Through Suppressing IL-32. Front. Pharmacol..

[29] Bai Y., Chen D., Cheng C., Li Z., Chi H., Zhang Y., Zhang X., Tang S., Zhao Q., Ang B. (2022). Immunosuppressive landscape in hepatocellular carcinoma revealed by single-cell sequencing. Front. Immunol..

[30] Kurtulus S., Sakuishi K., Ngiow S.F., Joller N., Tan D.J., Teng M.W., Smyth M.J., Kuchroo V.K., Anderson A.C. (2015). TIGIT predominantly regulates the immune response via regulatory T cells. J. Clin. Investig..

[31] Fourcade J., Sun Z., Chauvin J.-M., Ka M., Davar D., Pagliano O., Wang H., Saada S., Menna C., Amin R. (2018). CD226 opposes TIGIT to disrupt Tregs in melanoma. JCI Insight.

[32] Chen X., Xue L., Ding X., Zhang J., Jiang L., Liu S., Hou H., Jiang B., Cheng L., Zhu Q. (2022). An Fc-Competent Anti-Human TIGIT Blocking Antibody Ociperlimab (BGB-A1217) Elicits Strong Immune Responses and Potent Anti-Tumor Efficacy in Pre-Clinical Models. Front. Immunol..

[33] Mito I., Takahashi H., Kawabata-Iwakawa R., Ida S., Tada H., Chikamatsu K. (2021). Comprehensive analysis of immune cell enrichment in the tumor microenvironment of head and neck squamous cell carcinoma. Sci. Rep..

[34] Lecerf C., Kamal M., Vacher S., Chemlali W., Schnitzler A., Morel C., Dubot C., Jeannot E., Meseure D., Klijanienko J. (2019). Immune gene expression in head and neck squamous cell carcinoma patients. Eur. J. Cancer.

[35] Wu L., Mao L., Liu J.-F., Chen L., Yu G.-T., Yang L.-L., Wu H., Bu L.-L., Kulkarni A.B., Zhang W.-F. (2019). Blockade of TIGIT/CD155 Signaling Reverses T-cell Exhaustion and Enhances Antitumor Capability in Head and Neck Squamous Cell Carcinoma. Cancer Immunol. Res..

[36] Mao L., Xiao Y., Yang Q.-C., Yang S.-C., Yang L.-L., Sun Z.-J. (2021). TIGIT/CD155 blockade enhances anti-PD-L1 therapy in head and neck squamous cell carcinoma by targeting myeloid-derived suppressor cells. Oral Oncol..

[37] Kitsou M., Ayiomamitis G.D., Zaravinos A. (2020). High expression of immune checkpoints is associated with the TIL load, mutation rate and patient survival in colorectal cancer. Int. J. Oncol..

[38] Wen J., Mao X., Cheng Q., Liu Z., Liu F. (2021). A pan-cancer analysis revealing the role of TIGIT in tumor microenvironment. Sci. Rep..

[39] Xiao K., Li K., Xue P., Zhu S. (2021). Prognostic Role of TIGIT Expression in Patients with Solid Tumors: A Meta-Analysis. J. Immunol. Res..

[40] Preillon J., Cuende J., Rabolli V., Garnero L., Mercier M., Wald N., Pappalardo A., Denies S., Jamart D., Michaux A.-C. (2021). Restoration of T-cell Effector Function, Depletion of Tregs, and Direct Killing of Tumor Cells: The Multiple Mechanisms of Action of a-TIGIT Antagonist Antibodies. Mol. Cancer Ther..

[41] Kohli K., Pillarisetty V.G., Kim T.S. (2021). Key chemokines direct migration of immune cells in solid tumors. Cancer Gene Ther..

[42] Humblin E., Kamphorst A.O. (2019). CXCR3-CXCL9: It’s All in the Tumor. Immunity.

[43] Niu J., Maurice-Dror C., Lee D., Kim D.-W., Nagrial A., Voskoboynik M., Chung H., Mileham K., Vaishampayan U., Rasco D. (2021). First-in-human phase 1 study of the anti-TIGIT antibody vibostolimab as monotherapy or with pembrolizumab for advanced solid tumors, including non-small-cell lung cancer^☆^. Ann. Oncol..

[44] Van den Mooter T.F.A., Migeotte A., Jungels C., Delafontaine B.R., Nguyen T.L.-A., Warot S., Truong C., De Henau O., Driessens G., Lager J. (2021). Abstract CT118: Preliminary data from Phase I first-in-human study of EOS884448, a novel potent anti-TIGIT antibody, monotherapy shows favorable tolerability profile and early signsof clinical activity in immune-resistant advanced cancers. Cancer Res..

[45] Mettu N.B., Ulahannan S.V., Bendell J.C., Garrido-Laguna I., Strickler J.H., Moore K.N., Stagg R., Kapoun A.M., Faoro L., Sharma S. (2022). A Phase 1a/b Open-Label, Dose-Escalation Study of Etigilimab Alone or in Combination with Nivolumab in Patients with Locally Advanced or Metastatic Solid Tumors. Clin. Cancer Res..

[46] Shirasuna K., Koelsch G., Seidel-Dugan C., Salmeron A., Steiner P., Winston W.M., Brodkin H.R., Nirschl C.J., Abbott S., Kinugasa F. (2021). Characterization of ASP8374, a fully-human, antagonistic anti-TIGIT monoclonal antibody. Cancer Treat. Res. Commun..

